# Diffusion tensor imaging studies in vascular disease: A review of the literature

**DOI:** 10.1590/S1980-57642012DN06030008

**Published:** 2012

**Authors:** Gilberto Sousa Alves, Felipe Kenji Sudo, Carlos Eduardo de Oliveira Alves, Letice Ericeira-Valente, Denise Madeira Moreira, Eliasz Engelhardt, Jerson Laks

**Affiliations:** 1 Institute of Psychiatry, Universidade Federal do Rio de Janeiro, Rio de Janeiro RJ, Brazil; 2 Institute of Neurology, Universidade Federal do Rio de Janeiro, Rio de Janeiro RJ, Brazil; 3 Radiology Service of the Procardíaco Hospital, Rio de Janeiro RJ, Brazil; 4 Universidade Estadual do Rio de Janeiro, Rio de Janeiro RJ, Brazil

**Keywords:** diffusion tensor, DTI, neuroimaging, vascular disease, white matter, cognition, vascular cognitive impairment

## Abstract

Cerebrovascular disease (CVD) is often present in old age and may be associated with microstructural pathology of white matter (WM) and cognitive dysfunction. The current review investigated the relationship between CVD, cognitive status and WM integrity as assessed by diffusion tensor imaging (DTI).

**Methods:**

DTI studies were searched on ISI and Pubmed databases from 2002 to 2012.

**Results:**

Studies evidenced DTI changes in WM as associated with vascular disease and provide increasing support for DTI as a valuable method for early detection of CVD.

**Conclusion:**

DTI parameters can serve as important biomarkers in monitoring vascular disease progression and treatment response and may represent a surrogate marker of WM tract integrity.

## Abstract (pt)

A doença cérebro-vascular (DCV) está frequentemente presente na idade avançada, podendo se associar à patologia microscópica da substância branca (SB) e à disfunção cognitiva. A presente revisão investiga a relação entre DCV, status cognitivo e a integridade da SB, através da avaliação pelo tensor de difusão (DTI).

**Métodos:**

Os estudos em DTI foram selecionados a partir das bases ISI e Pubmed entre 2002 e 2012.

**Resultados:**

Os estudos evidenciaram alterações do DTI na SB associadas à doença vascular e fornecem evidencia importante para o DTI como um método valioso para a detecção precoce da DCV.

**Conclusão:**

Os parâmetros do DTI podem servir como importantes biomarcadores na monitoração da doença vascular quanto a sua progressão e resposta terapêutica e parecem representar um marcador substituto da integridade da SB.

## INTRODUCTION

Cerebrovascular disease (CVD) occurs in one third of the population^1^ and is often described as a pathological finding on brain Magnetic Resonance Imaging (MRI). Depending on the site, intensity, and severity, CVD may either cause or contribute to further cognitive decline.^2^ Earlier reports describe CVD pathology as a consequence of blood perfusion deficits generated by hypoxia, hypoperfusion and hemorrhage which in turn result in neuronal injury, necrosis, apoptosis and ischaemic penumbra.^2^ The presence and severity of CVD hinge on non-modifiable risk factors such as ageing, gender and genetics but also on several other variables such as smoking, systemic arterial hypertension, diet and metabolic diseases. Structural studies have identified subcortical hyperintensities as macroscopic white matter (WM) changes which have been cited by several reports as associated with CVD, mood disorders, executive dysfunction and higher conversion to dementia. More recently, the underlying pathology associated with normal-appearing WM as well as its clinical significance has become the main focus of investigation.^3^

In recent years, novel methods of neuroimaging have enabled WM microstructure integrity to be investigated *in vivo*.^4-6^ One of the most useful of these techniques is diffusion tensor imaging (DTI), which is sensitized to the motion of water molecules as they interact within tissues, thus reflecting characteristics of their immediate structural surroundings.^7,8^ Earlier DTI studies used a region-of-interest (ROI) analysis approach, with brain areas being delineated manually or with semi-automated methods.^9-11^ However the ROI approach has a number of drawbacks, such as difficulty precisely replicating and delineating anatomical regions as well as the use of pre-selected brain areas rather than considering diffusion changes in the whole brain.^12^ To improve the objectivity and interpretability of DTI studies, Tract-Based Spatial Statistics (TBSS) was developed to enable DTI scans to be compared across subjects more robustly.^13^ TBSS is based on voxel-wise analysis, which approaches the whole brain without any *a priori* selection of regions. Another advantage of TBSS is that it minimizes the problem of misalignment.^14^ To date, the vast majority of studies have concentrated on the role of FA in cognitive disorders.^5,15-17^ However, a range of factors influence FA decreases, including myelination, axon density, axon diameter and intra-voxel coherence of fiber orientation.^18,19^ Therefore, there is an increasing awareness of the limitations of single FA measurements, and of the need to investigate how other DTI indices (such as axial diffusion, mean diffusivities and radial diffusion) change over the course of both vascular and neurodegenerative diseases.^6,15^

There is an open field of investigation on DTI and CVD. To date, the majority of studies conducted have investigated only WM abnormalities in relation to neurodegeneration predominantly among AD patients but the pathological processes of WM associated with vascular disease are not yet fully understood. In contrast to the damaging effects of CVD and untreated hypertension on diffusion-based parameters of WM,^2^ the influence of milder vascular risk factors such as controlled hypertension, or high normal blood pressure is largely unknown.^2^ Another promising topic of DTI investigation is the common pathological routes between CVD and Alzheimer's Dementia (AD), especially the landscape involving neurodegenerative and vascular changes. Cerebral atherosclerosis is associated with a higher risk of AD while cardiovascular risk factors are associated with clinically-diagnosed AD and vascular dementia (VaD).^2,20^ The similarities in association between cardiovascular risk factors and dementia diagnosed as AD or VaD underline the relevance of CVD for aging-related cognitive decline in general and the flaws in simplistic diagnostic categories.^20^ Although vascular and degeneration processes often overlap, relatively few studies have focused on the interaction between these two pathologies. Conversely, no serum or plasma biomarker has been established as a reliable biomarker of CVD compared to other types of dementia and non-demented individuals.^20^

This review investigated the main results of DTI studies in patients with CVD. We aimed to discuss these results and the integration of diffusion findings with structural data and WM microscopic pathology and progression of cognitive impairment in relation to vascular disease.

## METHODS

A systematic review of DTI studies on CVD was performed by searching data from ISI and Pubmed web databases from the first DTI studies in 2002 (January) to 2012 (May). The search strategy included key words aimed at investigating a broader spectrum of primary vascular disorders affecting subcortical areas, particularly white-matter lesions: DTI, vascular dementia, subcortical disease, white-matter, neuroimaging, dementia, MCI, blood vessels.

All abstracts were independently read by six authors (GSA; EE; FKS; CEOA; LEV; JL) and those studies which complied with the inclusion criteria were selected for further reading. A manual search was also performed to retrieve articles related to this subject found among the references of the selected studies. The articles which satisfied all the following criteria were included for further reading and analysis. For inclusion, articles had to:

[1] have at least one DTI parameter, such as FA;
[2] be cohort, cross-sectional, or case-control studies with at least one criterion for vascular dementia (DSM-IV, or NINDS-AIREN, or ICD-10, or ADDTC) and mild cognitive impairment, vascular type as developed by the NINDS research group;^21^
[3] provide data on cognitively impaired patients ≥60 years of age, with or without clinical diagnosis of dementia;^4^ and
[4] include a comprehensive neuropsychological assessment.

## RESULTS

The Pubmed electronic search retrieved 156 articles. Only 12 remained eligible for further analysis. Table 1 depicts the main characteristics of the selected studies. Five articles out of the 12 selected used a whole brain analysis approach whilst 7 used a region of interest (ROI)-oriented approach.

**Table 1:** DTI studies.

Authors	Sample (N)	DTI parameters	Anatomical and clinical findings
Shao-qiong et al.^45^	AD (10)AD-VD (10)Controls (10)	MD, FA	• AD versus AD-VD: AD group differed from Vascular Group in MD values.
Burgmans et al.^32^	96 volunteers (36 with HBP); age range 50-77 y	FA, ADC	• Hypertension independently associated with low FA and exacerbating age differences in FA. • No differences in ADC among the three groups or in FA histograms between Post-stroke CN and CN groups.
Zhou et al.^46^	VCI (19)Post-Stroke CN (19)CN (19)	FAADC	• Mean FA significantly lower in the VCI in comparison with Post-Stroke CN and CN groups. • FA histogram peak height was higher in the VCI group and correlated with MMSE scores.
Vernooij et al.^27^	Non-demented subjects (832)	FA, AD, RD	• Decrease of FA in the periventricular regions was associated with WMH. • Low FA correlated with WM atrophy in the CC, fornix and the cingulate bundle up to its connection to the hippocampal region. • An increase in AD and RD in the fornix and hippocampal regions was associated with WM atrophy. • An increase in AD in the ventricles extending to the centrum semiovale and corona radiata was correlated with WMH burden.
Fu et al.^22^	AD (20) SIVD (20)Controls (20)	FA, ADC	• SIVD versus controls: SIVD showed lower FA and higher ADC in the IFOF, CC (genu and splenium) and SLF.AD versus controls and SIVD: • AD showed lower FA in the frontal and temporal lobes, hippocampus, IFOF, CC and higher ADC in the temporal lobe and hippocampus. • SIVD versus AD: SIVD showed higher ADC in the IFOF, CC and SLF; and lower ADC in the temporal lobe and hippocampus.
Shim et al.^26^	Non vascular MCI (21)Vascular MCI (19)controls (17)	FA	• Both vascular and non-vascular MCI versus controls: lower FA and higher MD in the frontal, parietal and temporal cortices; lower FA in the CC and frontal and temporal cortices. • Vascular MCI versus non-vascular MCI : vascular MCI showed lower FA in the parietal cortex and centrum semiovale;vascular MCI had higher FA in the hippocampal WM tracts
Kennedy et al.^29^	controls (77)	FA, ADC	• Hypertension modified the effect of age in the WM in the occipital and temporal lobes.
Otsuka et al.^20^	Individuals with extensive HDWM (24)	FA, MD	• FA reduction and MD increases in both the corpus callosum and HDWM correlated with MMSE score deterioration.
O'Sullivan et al.^24^	SIVD (36)Controls (17)	FA, MD	• MD increases in the centrum semiovale and anterior periventricular regions correlated with executive tasks; No clear pattern of correlation between FA and WMH burden.
Schimidt et al.^31^	Patients with extensive leukoaraiosis (340)	ADC	• WMH burden correlated with ADC histogram of whole brain tissue, normal-appearing brain tissue. • Memory, executive function and speed correlated with global mean ADC; associations with mean ADC of WMH burden were marginal and limited for memory and speed.
Vernnooij et al.^28^	Non-demented patients (860)	FA, MD	• Regardless of WMH burden, a higher MD or AD and RD was independently associated with worse performance on processing speed and global cognition.
Salat et al.^39^	Healthy older adults (128)	FA, AD, RD	• Bilateral regional associations between MABP and FA and radial diffusivity in several large portions of frontal and parietal WM tracts. Effects of blood pressure were independent of age and remained in the anterior CC after controlling for WMH burden.

AD: axial diffusivity; ADC: apparent diffusion coefficient; CC: corpus callosum; CN: cognitively normal; FA: fractional anisotropy; IFOF: inferior frontal-occipital fasciculus; HBP: high blood pressure; HDWM: hemispheric deep white matter; MABP: mean arterial blood pressure; MCI: mild cognitive impairment; MD: mean diffusivity; MMSE: mini-mental state exam; RD: radial diffusivity; SLF: superior longitudinal fasciculus; SIVD: subcortical ischaemic vascular disease; VCI: vascular cognitive impairment; WM: white matter; WMH: white matter hyperintensities.

## DISCUSSION

The majority of studies evidenced diffusion changes in WM as associated with vascular disease and supported DTI as a valuable method for the early diagnosis of CVD.^22^ The classical view in which vascular disease was related to WM hyperintensity (WMH) burden has been progressively substituted by new insights on the pathological development of brain vascular disease and its dynamic interaction with neurodegeneration. DTI papers support previous evidence from genetics and biomarkers^23-25^ showing that vascular disease plays an important role in the development and clinical course of AD.^26^ The ensuing topics cover aspects of the pathology and clinical features of the studies.

**The pathological basis of vascular changes.** There is growing evidence from DTI studies demonstrating that macroscopic and microscopic changes in WM result from distinct pathological processes which have been described by some authors as WM lesion formation and WM atrophy.^27^ Both processes result from a complex combination of independent factors such as age, hypertension, metabolic and degeneration, but there is no established model explaining these underlying changes.^27,28^

The territorial pattern of progression of WM changes has been discussed in some papers with equivocal findings. Parietal WM and the centrum semiovale were reported as the regions most associated with the vascular pathology^26^ whereas temporal and occipital lobes were described by another study.^29^ In a third study, periventricular areas were described as more susceptible to ischemic injury.^27,30^

Among the WM tracts most susceptible to damage due to atrophy of WM were those components of the limbic system (which comprise the anatomic substrate of Alzheimer's disease) such as the fornix, the cingulum tracts and in the region of the hippocampus.^27^ Additionally, one study showed that atrophy of the corpus callosum was significantly associated with changes in diffusion in deep WM hyperintensities. According to Schimidt et al.,^31^ such findings can further etiologic understanding of age-related WM damage because they argue against a diffuse pathological process as the origin of WMH.

**Correlation of WM changes with clinical variables.** Diabetes and hypertension^32^ have been cited as the most important clinical variables associated with CVD. Hypertension has been associated with reduction in WM volume^29,33-35^ and increase in WMH burden.^29,36-38^ In a recent investigation,^39^ the effects of Mean Blood Pressure were present both in subjects with mild cardiovascular risk and those with established hypertension. Hence, significant effects of cognitive decline were present even in individuals outside the hypertensive range.^39^ Taken together, these findings support that systemic vascular function is an important variable to be considered in the investigation of cognitive changes also in the context of patients without known vascular disease or overt brain vascular changes.

**Use of overlapping DTI indices in the differential diagnosis between vascular disease and neurodegeneration.** Contrasting with earlier reports, recent evidence in vascular disease has attempted to investigate WM pathology through the combination of FA and non FA indices^5,6,15^ thus following a general tendency seen in other studies, e.g. those with Alzheimer individuals.^6,15,40^ The overlap between axial and radial diffusion increases was associated with atrophy of WM in different regions (Table 1) and could not be observed in WMH areas, which were associated only to increased axial diffusion. Studies using animal models^41,42^ have shown that degradation of myelin is related to increased diffusion perpendicular to the tracts (radial diffusion), while acute axonal injury results in a decrease in diffusion parallel to the fibers (axial diffusion).^27^ These increases suggest decreased packing within a voxel.^43^ An alternative explanation is that apparent increases in axial diffusion may stem from a loss in fiber coherence among regions with fiber crossing.^27,44^ Early reports characterize the vascular pathology of subcortical areas by extensive occlusion of arterioles and micro-atherosclerosis. One study^28^ showed statistically significant FA and MD differences in areas of WMH burden and those with apparent normal WM. FA-MD and MD-DR overlaps may thus reflect demyelination and axonal loss within the fibers and early vascular disease. However, as the interpretation of multiple indices is not clearly established, further studies should comprehensively analyze the application of DTI indices to the understanding of complex interactions between vascular disease and degeneration.

**Correlation between DTI and neuropsychological testing.** One outpatient study^20^ analyzed the association of brain structural parameters and cognitive tasks and found a greater coefficient of correlation with diffusion indices than with WM volume or WM burden rated by the Fazekas scale.^20^ In a large multicenter study conducted by the LADIS multicenter group,^31^ which included 340 patients with varying degree of macroscopic WMH (leukoaraiosis), the correlation of microscopic changes in normal-appearing brain tissue was more strongly related to executive and memory impairment than WM volume and WMH burden. A similar conclusion was found by the Rotterdam study (Table 1) in which, regardless of WMH severity, there was a stronger association between non-FA indices and performance on tasks of information, global cognition and processing speed (Table 1).^28^ Findings from these three studies^20,28,31^ evidence a greater variability of WM microscopic pathology and support non-FA indices as sensitive biomarkers for assessing the integrity of WM independently of other structural measurements such as atrophy of white and gray matter or WM burden. Future studies should evaluate the usefulness of multiple diffusion parameters as biomarkers of cognitive decline in research and their applicability in monitoring responses to therapeutic interventions and treatment success.

This summary of findings demonstrates that loss of integrity in normal-appearing WM points to an independent process of WMH burden and may reflect a constellation of pathophysiological interactions, including ageing, neurodegenerative mechanisms and also vascular disease. Current evidence highlights the increasing importance of vascular health as a major component of general neural aging as demonstrated by previous studies.^39^ DTI parameters can serve as important biomarkers in monitoring vascular disease progression and treatment response and may represent a surrogate marker of WM tract integrity. Therefore, the studies discussed in the current review encourage the use of multiple diffusion indices as important tools for the diagnosis of microscopic changes in WM associated with early-onset vascular disease.
